# The effect of periodontal therapy on glycemic control and fasting plasma glucose level in type 2 diabetic patients: systematic review and meta-analysis

**DOI:** 10.1186/s12903-016-0249-1

**Published:** 2016-07-30

**Authors:** Amare Teshome, Asmare Yitayeh

**Affiliations:** 1 Department of Dentistry, College of Medicine and Health Sciences, University of Gondar, Gondar, P.O. Box 196, Ethiopia; 2 Department of Physiotherapy, College of Medicine and Health Sciences, University of Gondar, Gondar, Ethiopia

**Keywords:** Periodontal disease, Type 2 diabetic patients, Periodontal therapy, HbA1c, Glycemic control, Fasting plasma glucose, Scaling and root planning

## Abstract

**Background:**

Diabetic mellitus and periodontal disease have bilateral associations. However, there is a dilemma on the effect of periodontal therapy on glycemic control and/or fasting plasma glucose level in type 2 diabetic patients with periodontitis. Therefore, this review aimed to assess the effectiveness of periodontal therapy versus no periodontal therapy on glycated hemoglobin (HbA1c) and fasting plasma glucose level in type 2 diabetic patients.

**Methods:**

Article searching was done using four databases (MEDLINE, Cochrane library (CENTRAL), EMBASE and CINAHL) and a manual search (until December 2015). We included randomized controlled trials testing the effectiveness of periodontal therapy on glycated hemoglobin and fasting plasma glucose level in patients with type 2 Diabetes mellitus with periodontal disease. Studies published in English between 2005 and 2015 were included. Risk of bias was assessed regarding randomization, allocation sequence concealment, blinding, incomplete outcome data, selective outcome reporting, and other biases.

**Results:**

After the article selection process, seven Randomized controlled trials involving 940 participants with a primary outcome of change in glycated hemoglobin and/or fasting plasma glucose and having a minimum of 3 months follow-up were included.

There was a reduction of glycated hemoglobin 0.48(95 % CI: 0.18–0.78) after 3 months follow-up and 0.53 (95 % CI: 0.24–0.81) at the end of the intervention period.

There was also a significant reduction of fasting plasma glucose level, 8.95 mg/dl (95 % CI: 4.30–13.61) in the intervention group after the end of the intervention. The pooled analysis showed that patients with adjunctive antibiotic therapy and mouth wash had effect size of 0.51(0.03, 1.00, *p* = 0.04) and it was 0.53 (95 % CI: 0.19, 0.87; *p* = 0.002) in patients without adjunctive therapy. The publication bias of the studies was 0.066 according to Egger’s test.

**Conclusion:**

In this systematic review and meta-analysis, there is a significant reduction of Glycated hemoglobin and Fasting plasma glucose level on type 2 diabetic and periodontal patients with non-surgical periodontal therapy.

## Background

Periodontal disease is a chronic bacterial infection of the gingival, Periodontium, and the bone that support the tooth, which is caused by gram-negative anaerobic microorganisms that adhere to teeth as a bacterial plaque [1]. The frequency and severity of periodontitis are more in patients with systemic diseases (HIV/AIDS, diabetes mellitus (DM), and cardiovascular disease) and on pregnancy than healthy individuals [2].

Periodontal disease and DM are the two major chronic diseases which have a devastating effect on the health and wellbeing of millions of individuals globally. The prevalence of periodontitis is 10 % to 15 % of adult healthy patient [3]. However, type 2 DM patients have 2.8 times affected by periodontitis [4] and 4.2 times more likely to have alveolar bone loss than non-diabetic patients [4] . An emerging body of evidence has been reported that periodontal disease causes poor glycemic control and induced diabetes-related complications [5, 6].

Observational studies consistently showed that DM is one of the risk factors for the severity and progression of gingivitis and periodontal disease [7–12]. On the other hand, periodontal disease may be a possible risk factor for poor glycemic control and promote the existence of diabetic complications [9, 13]. In addition, it has been showed that periodontal disease has a devastating effect on glycemic control among type 2 diabetic patients and a significant reduction of Glycated hemoglobin(HA1c), 0.40 %, was observed after 3–4 months of periodontal therapy done on patients with both type 1 and 2 DM and periodontitis [14].

Impairment of glycemic control in diabetic patients can cause a decline in polymorphonucleate leukocytes activity. It can also damage the micro vascular endothelium which as a result can cause periodontal disease [15]. Diabetic patients with severe periodontitis are six times to have poor glycemic control than patients with healthy periodontium [16]. However, improved glycemic control has been postulated to reduce the severity of periodontal disease [16].

Non-surgical periodontal therapy (mechanical removal of pathogenic bacteria from the periodontium using Scaling and root planning (SRP) technique) has a beneficiary effect on the healing process of the periodontal tissue in healthy individuals. However, the healing process of the periodontium on diabetic patients depends on the level of glycemic control. The presence of a positive association between periodontal therapy and glycemic control has a significant clinical impact because every 1 % reduction in HbA1c significantly reduces the risk of diabetic complications [17].

Some literatures supported the hypothesis of periodontal therapy has a positive effect on glycemic control, which in turn reduces the incidence of diabetic related complications [18–21]. However, some studies showed there is no association between periodontal therapy and glycemic control in diabetic patients [22–25]. Due to this inconsistency in literature, there is conflict on clinical aspects to make a clinical recommendation with confidence.

Therefore, this systematic review and meta-analysis is intended to answer the following question: in type 2 diabetic patients with periodontal disease, does periodontal therapy (compared to no therapy) have a reduction on the effect of glycemic and fasting plasma glucose level?

## Methods

### Protocols and registration

The systematic review was done using the preferred reporting items for systematic reviews and meta-analysis (PRISMA) checklist. There was no registration done either for the protocol or the systemic review.

### Eligibility criteria

The types of included studies were Randomized controlled trials (RCTs) which studied the effectiveness of periodontal therapy on glycemic control and/or fasting plasma glucose level in type 2 DM with periodontitis. Full text articles published in English (as translation funding was not available), done on type 2 diabetic patients with periodontitis, periodontal treatment with/without adjunctive treatment, and outcome of change in glycated hemoglobin and/or fasting plasma glucose.

### Information sources

Studies were identified by searching electronic databases (MEDLINE, EMBASE, CINAHL and Cochrane Library (CENTRAL)) and scanning reference lists of articles and using manual search. Articles published in English language were selected. The search was started in May 2015 and ended in December 2015 for all databases.

### Data search

Four databases (were used during article selection process from their inception to December 2015. In addition to this, a manual search of articles was done from following journals; Dental research journal, Journal of dental research, Journal of Clinical Periodontology, Journal of periodontal research, Journal of the American dental association, Clinical Diabetes, Diabetes Care, and Diabetes research and Clinical Practice.

We used the following search keywords to search all clinical trials registers and databases: “periodontal therapy and type 2 diabetes; periodontal disease and type 2 diabetes mellitus; periodontal disease and glycemic control; periodontal disease and Diabetes mellitus.

### Article selection

The two authors searched articles electronically in databases and manually. To identify relevant articles; titles and abstracts of retrieved papers were exported to Endnote where duplicates were identified and removed by one reviewer (AT).

**Fig. 1:**
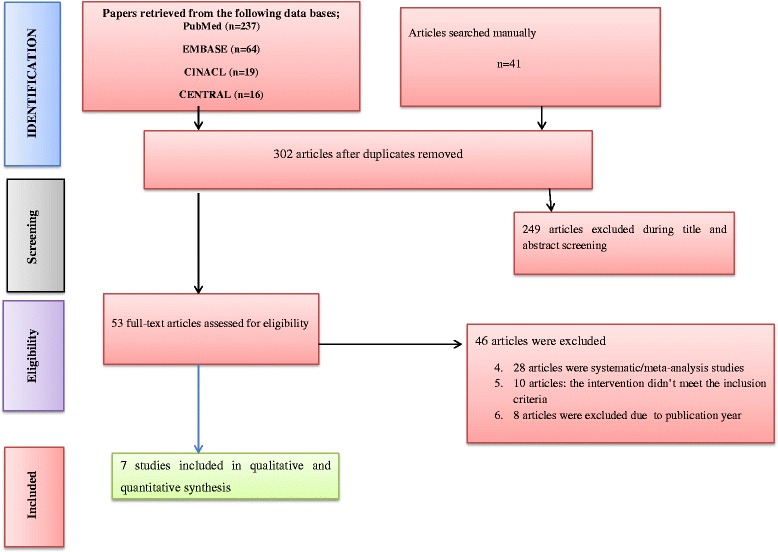
Flow diagram showing the article selection process

All retrieved articles were evaluated by two independent reviewers (AT, AY). A conflict between these two reviewers was resolved by consensus. After excluding the non-eligible articles, they were screened according to their titles and abstracts. Full-text articles were assessed and the articles which didn’t meet the inclusion criteria were excluded (Fig. 1).

### Type of intervention

The intervention groups receive non-surgical periodontal therapy with/without adjunctive antibiotics or mouth wash while the control groups didn’t receive periodontal therapy until the end of the intervention period (Table 1).

**Table 1:** Characteristics of studies included in the meta-analysis and change in HbA1c and FPG level

Author/publication year	Country	Sample size	Mean age	Inclusion criteria of diabetes, initial HbA1c (%), diabetes duration (Year)	Inclusion criteria of periodontal disease	Interventions	Duration of intervention	Outcome measures	Results
									∆HbA1c(%)/FPG Level
Koromantzos et al., 2011 [36]	Greece	I = 30 C = 30	I = 59.62 ± 7.95 C = 59.42 ± 9.8	Type 2DM A1C levels (7 % −10 %) Initial HbA1c: I = 7.0–9.9 C = 7.0–10.2 Diabetes Duration I = 7.76 ± 4.33 C = 7.84 ± 6.80	having at least 16 teeth present with at least eight sites (PPD) >6 mm and four sites with CAL >5 mm, distributed in at least two different quadrants.	I = SRP + OHI C = delayed treatment	6 months	HbA1c	HbA1c Changes within the 1st 3 month I: 0.73 ± 0.66 C: 0.18 ± 0.59 HbAIc changes after 6th months I: 0.72 ± 0.93 C: 0.13 ± 0.46
Kumar et al., 2015 [30]	India	I = 15 C = 15	NA	Type 2 DM HbA1c level 7.5–11 % Initial HbA1c: I = 8.13 ± 0.67 C = 8.09 ± 0.76 FPG (mg/dl) base line I = 153.63 ± 13.02 C = 157.35 ± 17.78	1, Chronic generalized periodontitis patients (Armitage Criteria) 2. Patients who have undergone periodontal treatment 6 months prior to the study	I = SRP and systemic Doxycycline C = no treatment	3 months	HbA1c	HbA1c Changes after 3rd month I = 7.31 ± 0.50 C = 8.16 ± 0.74 Change in FPG after three months I = 145.47 ± 11.17 C = 157.93 ± 17.44
Kanduluru and Naganandini, 2014 [32]	India	I = 35 C = 35	I = 47.14 ± 5.28 C = 48.86 ± 3.81	Type 2DM HbA1c level Initial HbA1c: I = 8.49 ± 1.50 C = 8.04 ± 0.70 Initial FPG Level I = 131.31 ± 21.48 C = 127.34 ± 19.89 Diabetes duration I = 1–15 years C = 1–15 years	1. pocket depth [PD] 4–6 mm involving >30 % sites) 2. generalized moderate periodontitis	I = SRP + OHI C = OHI	3 months	HbAIc FBS	HbA1c Changes after 3rd month I = 8.47 ± 0.89 C = 8.27 ± 0.63 FPG changes after 3rd months I = 122.64 ± 17.81 C = 136.57 ± 15.07
Engebretson et al., 2013 [34]	USA	I = 257C = 257	I = 56.7 ± 10.5 C = 57.9 ± 9.6	Type 2Dm HbA1c level 7.0 % −9.0 % Initial HbA1c: I = 7.84 (0.65) C = 7.78 (0.60) Baseline FPG I = 150 (125–174) C = 147 (122–172) Diabetes duration I = 12.3 (8.2) C = 11.3 (8.4)	1. a minimum of 16 natural teeth 2. CAL/PD >5 mm in 2 or more quadrants of the mouth 3. No periodontal treatment in the prior 6 months.	I = SRP + OHI chlorhexidine gluconate, toothbrush, toothpaste, Dental floss. C = OHI	6 month	HbA1c FBS	Mean Change of HbA1c (95 % CI) at 3rd month I = 0.14(0.02–0.27) C = 0.11(−0.02 to 0.24) Mean Change of HbA1c (95 % CI) at the end of 6th month I = 0.15(−0.01 to 0.30) C = 0.09(−0.06 to 0.25)
Gay et al., 2014 [35]	USA	I = 66 C = 60	I = 51.5( 9.0) C = 54.0(10.2)	1. Type 2DM HbA1c level 4.0–15.0 % Initial HbA1c: I = 9.0 ± 2.3 C = 8.4 ± 2.0 2. nonsmokers	1. no systemic antibiotic therapy within 6 months of recruitment, 2. the presence of localized or generalized severe chronic periodontitis	I = SRP + OHI C = OHI	4 months	HbA1c	HbA1c Changes after 4th month I = 8.4 ± 1.9 C = 8.1 ± 1.8
Kaur et al., 2015 [31]	India	I = 50 C = 50	I = 51.82 ± 5.85C = 52.94 ± 6.03	1. Type 2DM Initial HbA1c: I = 8.17 ± 2.49 C = 7.87 ± 2.56 2. Duration of DM I = 8.57 ± 6.39 C = 7.05 ± 4.43 FPG (mmol/L) I = 137.12 ± 59.71 C = 120.74 ± 41.47	1. presence of ≥12 teeth 2. ≥2 interproximal sites, not on the same tooth, with an attachment loss ≥4 mm, or PD ≥5 mm. 3. ≥2 interproximal sites, not on the same tooth, with an attachment loss ≥6 mm, and one or probing depth ≥5 mm	I = OHI + SRP C = no treatment	3,6 months	HbA1c	HbA1c Changes in the end of 3rd month. I = 7.49 ± 1.83 C = 7.96 ± 2.65 HbA1c Changes in the end of 6th month. *I =* 7.29 ± 1.61 *C =* 8.06 ± 2.72 Change in FPG after three months I = 135.88 ± 59.54 C = 121.71 ± 42.39 Change in FPG after six months. I = 132.10 ± 56.91, C = 122.75 ± 42.67
Telgi et al., 2013 [33]	India	I = 20 C = 20	NA	1. Type 2 DM Initial HbA1c: I = 7.68 ± 0.63 C = 7.74 ± 0.59 2. blood sugar controlled only with oral hypoglycemic agents FPG(mg/dl) I = 210.50 ± 4.98 C = 210.5 ± 41.98	1. the presence of aminimum of 28 teeth 2. pocket depth of 4–5 mm) 3. no systemic antibiotic administration and no periodontal treatment in last six months	A = Scaling+ mouthwash+ brushing C = brushing	3 month	HbA1c	HbA1c after 3 month I = 7.10 ± 0.64 C = 7.75 ± 0.48 FPG after three month I = 181.70 ± 41.28 C = 210.1 ± 43.02

### Risk of bias assessment with in the studies

**Fig. 2:**
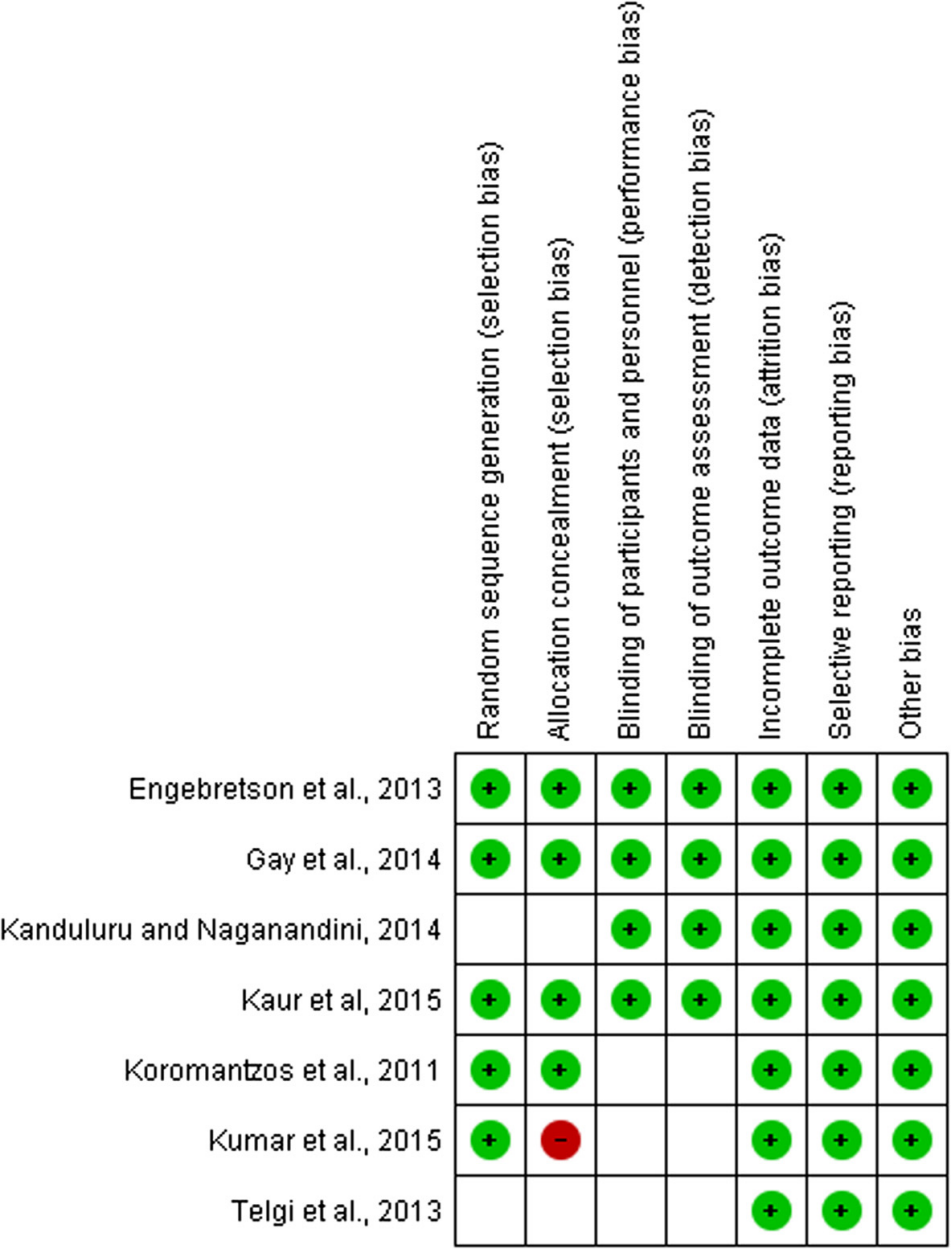
Risk of bias assessment table within studies

The two reviewers (AT, AY) assessed the risk of bias of the individual articles independently. The risk of bias of each included trial was assessed both at the study and outcome level with the following parameters; random sequence generation, allocation concealment, blinding of the examiner and/or patient, incomplete data outcome and loss to follow-up [26]. These parameters were assessed using the standardized form prepared by the Cochrane group [18] (Fig. 2 ).

### Data extraction

The two reviewers (AT, AY) extracted data from the selected randomized controlled trial (RCT) studies using pre-designed forms independently. Any conflict between these two reviewers was resolved by consensus. Data was extracted from each RCT on (1) demographic characteristics (including mean age, sample size, country of the study done); (2) definition of periodontitis; (3) definition of diabetes (type 2DM, duration, Initial and Final results of HA1c, initial and final results of fasting plasma glucose (FPG) level); (4) type of intervention and (5) the type of outcomes (glycated hemoglobin/fasting plasma glucose) (Table 1).

Change in HbA1c between baseline and final result after periodontal therapy was the primary outcome of the study. The difference in HbA1c percentage in each treatment was recorded. When there is no documented difference, it was calculated by extracting the mean change of glycated hemoglobin in the experimental and control group.

Change in FPG between baseline and final result after periodontal therapy was the second outcome of the study. The mean difference of FPG was registered in each treatment follow-up (Table 1).

### Statistical analysis

All data extracted from the studies were submitted and analyzed using RevMan 5.3 software [27]. The Meta analysis was performed, comparing the experimental group who got periodontal therapy and the control group which didn’t get periodontal treatment. From the selected studies, mean difference was calculated using random effect model. The heterogeneity of the studies was assessed by I-square. The publication bias of the articles was assessed using GRADE, funnel plot and Egger’s test.

## Results

### Study selection

A total of 367 articles were retrieved using four electronic databases (MEDLINE, EMBASE, CINAHL and Cochrane library) and manually searching. After removal of the duplicates 302 articles were remained. Out of those 249 articles were excluded during title and abstract screening, because these papers did not meet the inclusion criteria. Two independent reviewers (AT, AY) have scanned these journals using the predesigned form based on the title and the abstract of the paper. Fifty three full-text Articles were selected and assessed in more detail for eligibility. Seven articles met the inclusion criteria and were included in the systematic review and Meta-analysis (Fig. 1).

### Study characteristics

#### Methods

All seven studies selected for the Meta- analysis were randomized controlled trials published in English. Four of them were done in India [28–31], two of them were in USA [32, 33], and the remaining one was conducted in Greece [34]. All of the studies included patients having periodontitis and type 2 DM as study subjects (Table 1).

#### Participants

The included studies involved 473 participants (range 15–257 patients per study) in the intervention group and 467 participants (range 15–257 patients per study) in the control group. The mean age ranges from 47.14(±5.2) to 59.62(±7.95) years in the intervention group and from 48.86 (±3.8) to 59.42(±9.8) in the control group. The baseline level of HA1c ranged from 7.0 to 9.9 % in the intervention group while it was ranged from 7.0 to 10.2 % in the control group (Table 1).

#### Intervention

All patients in the intervention group received scaling and root planning with systemic doxycycline [28], mouth wash [31, 32] and without adjunctive antimicrobials or mouth wash [29, 30, 33, 34] while the control group received any form of periodontal intervention (Table 1).

#### Outcomes

In all studies the primary outcomes assessed were change in HA1c level and change in fasting plasma glucose level after the intervention level.

#### Risk of bias within studies

To ascertain the validity of eligible randomized controlled trials the two reviewers assessed the risk of bias of the selected studies using the following parameters.

Random sequence generation, allocation concealment, blinding of the personnel and participants, intention to analysis, incomplete outcome data addressed and selecting reporting. All of the articles reported status of incomplete outcome data, intention to treat analysis and selecting reporting. Random sequence generation and allocation concealment were not clearly stated in two studies [32, 33] (Fig. 2).

#### Risk of bias across the studies

**Fig. 3:**
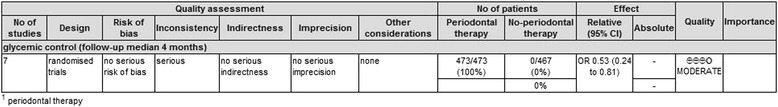
GRADE evidence summary for the assessment of risk of bias across the included studies

The risk of bias across the studies was assessed using GRADE profile and it was moderate as presented in Fig. 3.

**Fig. 4:**
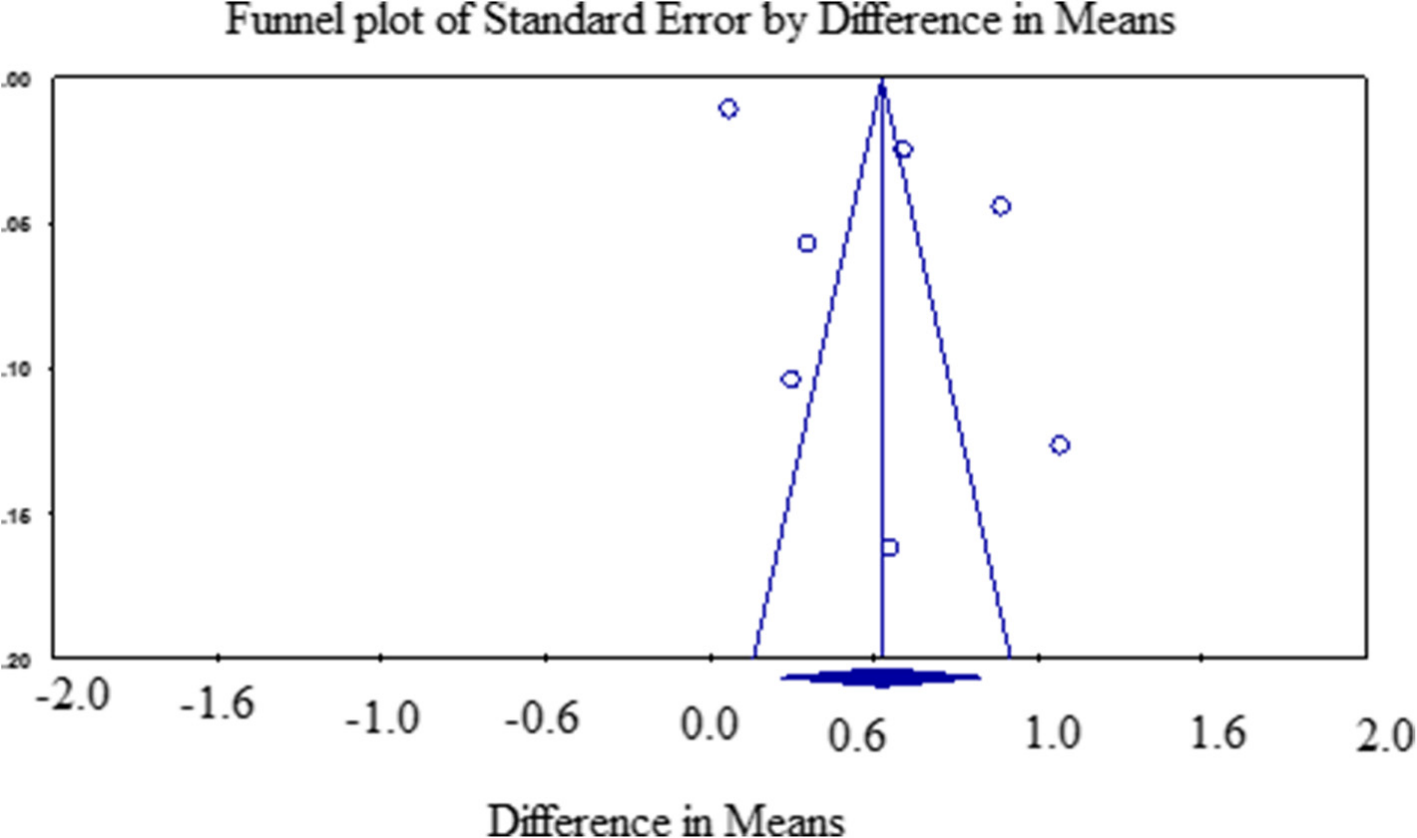
Funnel plot showing the distribution of the studies

We assessed the possibility of publication bias by evaluating a funnel plot of the mean difference for asymmetry. The funnel plot revealed an asymmetrical distribution of the included studies (Fig. 4), and the publication bias was 0.066 in Egger’s test.

#### Exploration of heterogeneity

In this random effects model, the heterogeneity (I^2^) among the selected seven studies is 99 % (*p* = 0.0003), suggesting the 99 % of the variability in treatment effect estimates is due to real study difference (heterogeneity) and only 1 % due to chance (Fig. 6).

#### Results of individual studies

The effect of periodontal therapy on glycemic control of type 2 diabetic patients was analyzed from the available randomized clinical trial studies. All of the studies showed a reduction in HA1c after three to four months follow-up [28–34] and there was a moderate difference of HA1c level between the baseline and end of the intervention. Four studies showed a change in FPG level from baseline to 3 months [28–31] (Table 1).

#### The effect of periodontal therapy on glycated hemoglobin (HbA1c)

**Fig. 5:**
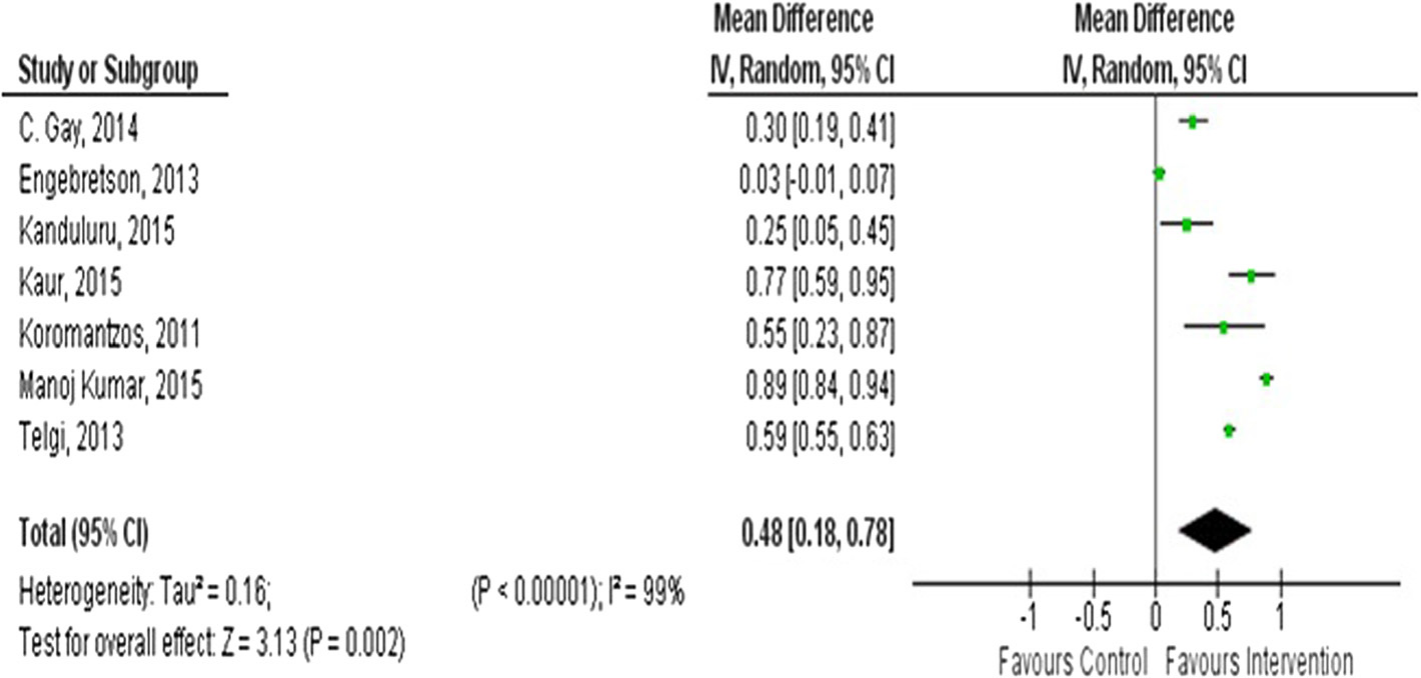
Meta-analysis showing the pooled effect of periodontal therapy on glycemic control at the end of 3 months follow-up

**Fig. 6:**
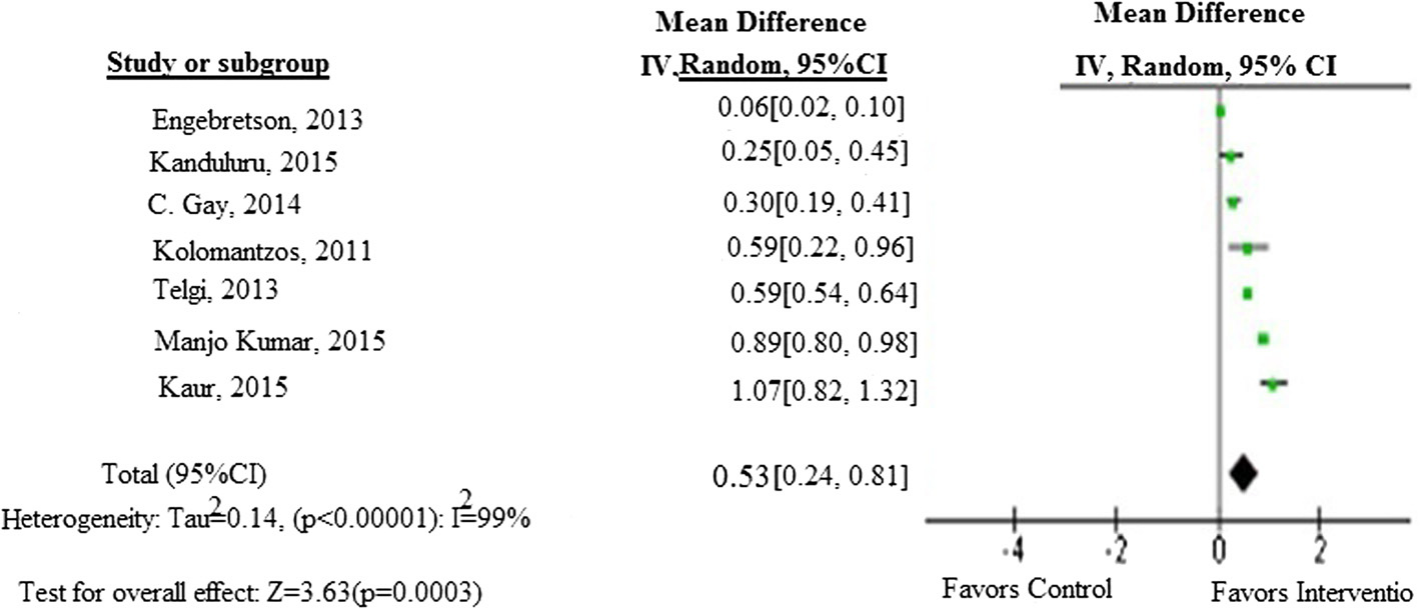
Meta-analyis showing the pooled effect of periodontal therapy on glycemic control at the end of the treatment

The mean difference in HA1c ranged from 0.02 (±0.61) to 0.88 (±0.66) in the intervention group and −0.23 (±0.07) to 0.3(±0.2) in the control group. A significant reduction of HbA1c was observed in the pooled analysis between the intervention and control group with a random effect of: 0.48 (95 % CI: 0.18, 0.78, *p* = 0.002) at the end of 3 months follow-up and a mean difference of 0.53 (95 % CI: 0.24, 0.81, *p* = 0.0003) with overall effect of Z = 3.63 after the end of the intervention period (Figs. 5 and 6).

**Fig. 7:**
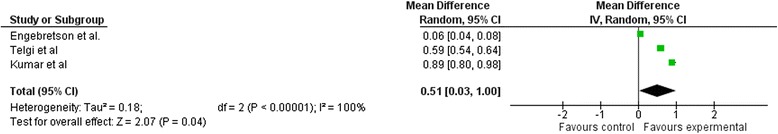
Meta-analysis showing the effect of adjunctive use of antibiotics and mouth wash along with periodontal therapy on glycemic control

**Fig. 8:**
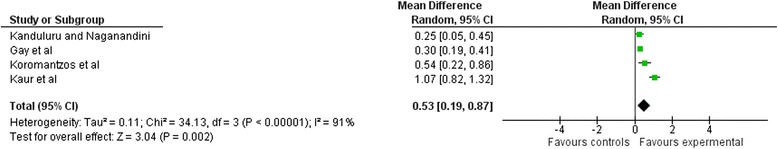
Meta-analysis showing the effect of periodontal therapy without adjunctive antibiotics/mouthwash on glycemic control on type 2 diabetic patients

Subgroup analysis of the studies was done depending on their intervention type (adjunctive antibiotic/mouth wash use or not). There was a statistically significant reduction of HA1c in the intervention group in those with adjunctive therapy: 0.51(95 % CI: 0.03, 1.00; *p* = 0.04) with 95 % heterogeneity and in non-adjunctive therapy: 0.53(95 % CI: 0.19, 0.87; *p* = 0.002) with 91 % heterogeneity (Figs. 7 and 8).

#### Periodontal treatment and fasting plasma glucose level

**Fig. 9:**
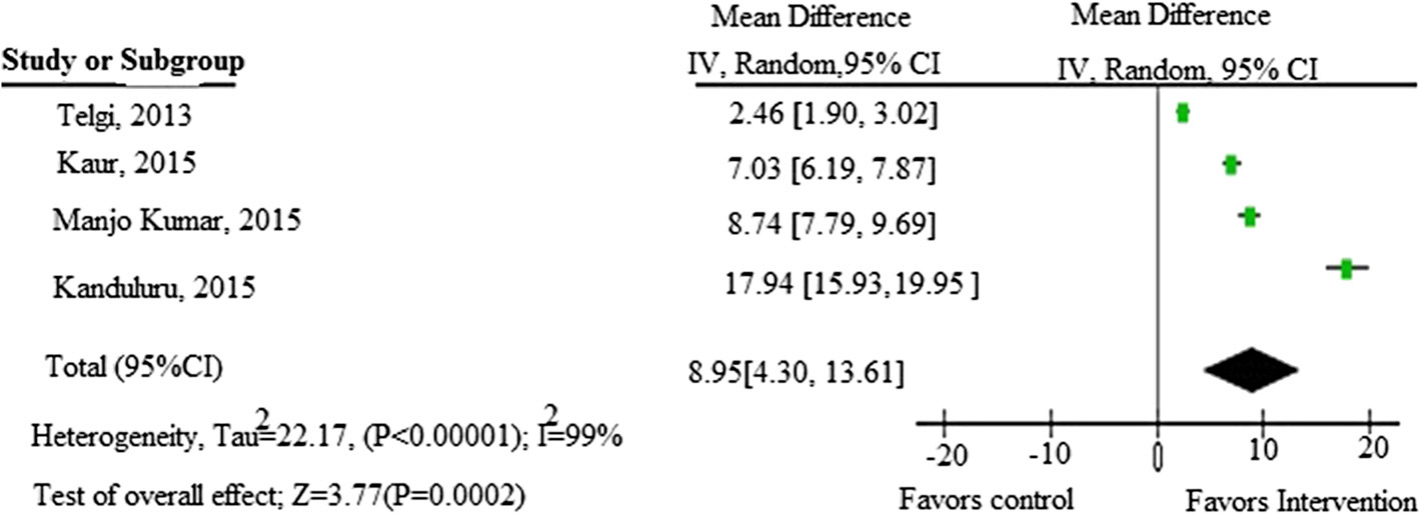
Meta-analysis showing the effect of periodontal therapy on fasting plasma glucose level reduction in type 2 diabetic patients with periodontitis

There was a mean difference of FPG level ranged from 5.02 (±2.8) mg/dl to-28.8 (±36.3) mg/dl in the intervention group while it was ranged from-9.23(±4.82) mg/dl to 0.4(±1.04) mg/dl in the control group. The effect of periodontal therapy on fasting plasma glucose was done in four studies. The computed mean difference was 8.95 mg/dl (95 % CI: 4.30, 13.61), favoring the intervention group (Fig. 9).

## Discussion

Uncontrolled diabetes mellitus has adverse effects on the health of the periodontium, which in turn, have a deleterious effect on the glycemic level of type 2 diabetic patient. The pathophysiology of this effect is associated with altered host response, inflammation, and insulin resistance [12]. In this meta-analysis, we analyzed data from seven randomized controlled trials, targeted at evaluating whether periodontal therapy had an influence on glycemic control in patients with both type 2 DM and periodontitis.

In this meta-analysis, the included articles comprised of a total population of 940 patients (473 patients in the intervention and 467 patients in the control group). It has large sample size than the previous studies [24, 35, 36]. The mean difference in HbA1c ranged from 0.02 (±0.61) to 0.88 (±0.66) in the intervention group and from −0.23 (±0.07) to 0.3(±0.2) in the control group. There was a mean difference of FPG level ranged from 5.02 (±2.8) mg/dl to-28.8 (±36.3) mg/dl in the intervention group while it was ranged from −9.23(±4.82) mg/dl to 0.4(±1.04) mg/dl in the control group.

Even though there were literature investigating the effect of periodontal therapy on glycemic control [18–23], there has not been consistence and complementary evidence on the effects of periodontal therapy on glycemic control and/or fasting plasma glucose levels. Some of the literature showed positive effect of periodontal therapy on glycemic control [18–21]. On the other hand, some researchers reported periodontal therapy has no effect on glycemic control besides its effect on periodontal health [22–25]. The present study is in favor of the hypothesis suggesting periodontal therapy has a beneficial effect on HbA1c and FPG reduction.

In the present meta-analysis, there was a moderate reduction of HA1c level in the intervention group, with a random effect size of 0.53 % HbA1c (95 % CI: 0.24, 0.81) among the seven studies which is statistically significant. This result is consistent with the results of the previous Meta-analyses done by Engebretson et al. (0.36 % (95 % CI −0.54, −0.19 %) [19], Teeuw et al. (−0.40 % (95 % CI −0.77, −0.04 %, *P* = 0.03) [37], Liew et al., (0.41 % (95 % CI: _0.73 %, _0.09 %,) [20], Darre’ et al. 0.46 (95 % CI 0.11, 0.82) [21] and Sun et al. (1.21 %(95 % CI: 0.75, 1.68) [35]. This comparable result indicates periodontal therapy has a moderate impact on the reduction of HbA1c after the treatment. However, a study done by Janket et al. (0.57 %, *p* = 0.82) [24] and Wang etal (−0.238, 95 % CI: −0.616 , 0.14; *p* = 0.217) [36] showed that there was a non-significant decrease of HbA1c between the intervention and the control group. This discrepancy might be due the small number of included studies and small sample size which may be insufficient to demonstrate significant differences [36].

In this meta-analysis, subgroup analysis of the studies was done depending on their intervention type (adjunctive antibiotic/mouth wash use or not). There was a statistically significant reduction of HA1c in both the intervention groups with adjunctive therapy; 0.51(95 % CI: 0.03, 1.00; *p* = 0.04) and non-adjunctive therapy: 0.53(95 % CI: 0.19, 0.87; *p* = 0.002). This result showed that the reduction of glycated hemoglobin depends on the presence of periodontal therapy and the presence of adjunctive antibiotic or mouth wash has no effect on glycemic control.

The result of this meta-analysis showed that periodontal therapy could lead to a significant reduction in FPG levels with effect size of 8.95 mg/dl (95 % CI: 4.30, 13.61; *p* = 0.0002) at the end of the intervention. This result is almost similar with the result of a study done by Wang et al. [36], which showed a significant decrease in FPG after 3–4 months of follow-up (−9.01 mg/dl (95 % CI −2.24 to −15.78; *p* = 0.009) and (−13.62 mg/dl, 95 % CI: 0.45 to −27.69) after 6 months (*P* = 0.06). However, a study done by Sun et al. [35] showed there was no significant difference in plasma fasting glucose level between the experimental and control groups after 3 months follow-up.

The limitation of this systematic review and meta-analysis was searching of literatures was restricted to full text articles published in English language due to financial problems. Majority of the included studies have a small sample size which could lead this systematic review and Meta-analysis to small-study effect. Variation in baseline HbA1c level among the studies and the presence of different periodontal definition and treatment period was observed in the selected studies which could bring an inconsistent result in the systematic review and Meta-analysis.

The other limitation of this systematic review with Meta- analysis is that some of the included articles didn’t report the standard deviation of change in FPG and HbA1c, and the data were obtained by calculation.

## Conclusion

The Meta analysis result supports the effectiveness of periodontal therapy in glycemic control in type 2 diabetic patients and periodontitis. However, large sample size Randomized controlled trials must be done to confirm this association.

### Clinical application

This result indicates that the use of periodontal treatment along with oral hypoglycemic drugs (agents) has a significant impact on glycemic control and/or fasting plasma glucose level. Based on this implication Type 2 DM patients with periodontitis are advised to have a regular periodontal treatment and follow up so that they can reduce the incidence of diabetic related complications.

It is advisable if dental and endocrinology departments have a bilateral link to prevent the diabetic related complications of those patients with periodontal disease and Type 2 DM.

## Abbreviations

DM, diabetes mellitus; FPG, fasting plasma glucose; HbA1c, glycated hemoglobin; OHI, oral hygiene instruction; PD, pocket depth; RCT, randomized controlled trials; SRP, scaling and root planning; USA, United States of America
